# Long-Term Air Pollution Exposure, Cardiovascular Disease, and Cardiometabolic Risk Factors in Middle-Aged and Older Chinese Adults: A Prospective Cohort Study

**DOI:** 10.3390/bioengineering13090958

**Published:** 2026-08-23

**Authors:** Xuehua Wang, Zhaohui Wang, Yan Wang

**Affiliations:** 1 Institute of Pathology, Tongji Hospital, Tongji Medical College, Huazhong University of Science and Technology, Wuhan 430030, China; 2 Department of Cardiology, Union Hospital, Tongji Medical College, Huazhong University of Science and Technology, Wuhan 430022, China; 3 Hubei Key Laboratory of Biological Targeted Therapy, Union Hospital, Tongji Medical College, Huazhong University of Science and Technology, Wuhan 430022, China; 4 Hubei Provincial Engineering Research Center of Immunological Diagnosis and Therapy for Cardiovascular Diseases, Union Hospital, Tongji Medical College, Huazhong University of Science and Technology, Wuhan 430022, China

**Keywords:** air pollution, cardiovascular disease, cardiometabolic risk factors, triglyceride-glucose index

## Abstract

The substantial burden of cardiovascular disease (CVD) attributable to air pollution remains a critical public health challenge, yet its combined effect with cardiometabolic factors is not fully understood. This study leveraged data from the Global Burden of Disease Study (GBD) and the China High Air Pollutants (CHAP) database to evaluate the CVD burden attributable to air pollution, which was found to increase progressively with age. We conducted a cross-sectional analysis using baseline (2011) data from 7420 participants aged ≥ 45 years in the China Health and Retirement Longitudinal Study (CHARLS), and a prospective cohort analysis in 8792 participants followed through 2020. In the longitudinal analysis, long-term exposure to PM_2.5_ (HR = 1.09, 95% CI: 1.03–1.16) and PM_10_ (HR = 1.07, 95% CI: 1.04–1.10) was significantly associated with an elevated risk of incident CVD, whereas the association for NO_2_ was nominally significant (HR = 1.12, 95% CI: 1.00–1.24) and that for O_3_ was not statistically significant (HR = 1.08, 95% CI: 0.93–1.27). Furthermore, prolonged exposure to these pollutants was associated with a higher prevalence of hypertension, diabetes, dyslipidemia, and obesity, and with adverse levels of metabolic indicators, including the TyG, TyG-BMI, and TyG-WC indices. A random forest model showed modest discrimination for incident CVD (AUC = 0.656), identifying PM_10_ and PM_2.5_ as the most important predictors. In conclusion, long-term exposure to ambient air pollution, particularly particulate matter, is associated with an increased risk of CVD and cardiometabolic disorders in middle-aged and older Chinese adults.

## 1. Introduction

Cardiovascular disease (CVD) represents a major global public health challenge, contributing significantly to morbidity, mortality, and economic costs worldwide [1,2]. It is estimated that 55 million deaths occurred worldwide in 2017, with almost one-third attributed to CVD [1]. The estimated years of life lost (YLL) due to CVD increased significantly [1]. The growing prevalence of moderate ischemic heart disease and ischemic stroke in China, compounded by an aging population and the suboptimal management of common conditions like hypertension, hyperlipidemia, and diabetes, is posing significant challenges for CVD prevention and treatment [3].

Air pollutants consist of a complex mixture of gaseous compounds (e.g., nitrogen oxides, ozone, sulfur dioxide, ammonia, carbon monoxide), volatile droplets (including quinones and polycyclic aromatic hydrocarbons), as well as both primary and secondary particulate matter (PM) [4]. PM comprises a complex and heterogeneous mixture of particles, which are typically classified based on their aerodynamic diameter into three fractions: coarse (PM_10_, <10 μm), fine (PM_2.5_, <2.5 μm), and ultrafine (PM_0.1_, <0.1 μm), respectively [5]. Ozone (O_3_) forms via photochemical reactions between ultraviolet radiation, nitrogen oxides (NO_x_), and volatile organic compounds (VOCs), acting as a harmful ground-level pollutant linked to respiratory/cardiovascular risks despite its protective stratospheric role [6]. In London, based on 2010 data, nearly 5900 premature deaths per year are linked to exposure to nitrogen dioxide (NO_2_), which is predominantly emitted by diesel-powered road vehicles [7]. The composition of air pollution varies based on environmental factors—such as geography and weather—and emission sources, including industrial operations, agriculture, and road, marine, and air transport [4]. Air pollution remains a major environmental risk in China, significantly contributing to both the national disease burden and economic losses. From 1990 to 2021, nationwide deaths attributable to air pollution rose by 3.38%, while the total disability-adjusted life years (DALYs) decreased by 28.63% [8]. Air pollution adversely affects multiple organs and systems throughout the body. In 2015, the World Health Organization passed a resolution addressing air quality and health, which formally recognized air pollution as a leading risk factor for numerous diseases, including ischemic heart disease, stroke, chronic obstructive pulmonary disease, premature death, and cancer [9]. However, research on the cardiovascular risks posed by multiple air pollutants remains scant, particularly among middle-aged and elderly populations.

The triglyceride-glucose (TyG) index is a composite measure derived from fasting triglyceride and fasting blood glucose levels. It is widely used as a reliable surrogate marker for insulin resistance (IR) and has been consistently associated with CVD [10]. Obesity and diabetes are well-established major risk factors for CVD. Accumulating evidence suggests that integrating the TyG index with conventional obesity indicators—such as body mass index (BMI) and waist circumference (WC)—enhances the prediction of cardiovascular risk [11]. Our study aims to investigate the associations of exposure to various air pollutants with CVD and its metabolic risk factors, as well as to develop a random forest-based model that integrates these exposures to predict CVD risk.

## 2. Materials and Methods

### 2.1. Data Collection

The Global Burden of Disease Study (GBD) is a comprehensive research program that uses a standardized methodology to quantify the burden of major diseases, injuries, and risk factors [12]. It provides annual estimates for 371 diseases and injuries from 1990 onwards, covering clinical outcomes for 3499 conditions across 204 countries and regions, including over 20 subnational areas in 20 countries. Data from the GBD were used to assess the burden of CVD.

### 2.2. Study Data and Population

The China Health and Retirement Longitudinal Study (CHARLS) is a nationally representative, longitudinal survey of community-dwelling adults aged 45 and older in China, designed to assess their social, economic, and health conditions. Its baseline wave (2011) used a multistage probability-proportional-to-size (PPS) sampling strategy, recruiting approximately 17,000 individuals from 10,000 households across 450 villages, 150 counties, and 28 provinces [13]. To date, CHARLS has collected five waves of data: the baseline in 2011 (wave 1), with follow-ups in 2013 (wave 2), 2015 (wave 3), 2018 (wave 4), and 2020 (wave 5). This study used data from all five waves (2011–2020). The initial sample consisted of 17,708 participants enrolled in the 2011 wave. After applying exclusion criteria, participants were excluded for the following reasons: (1) age under 45 years or missing age data; (2) missing data on CVD status; (3) missing data on core demographic and lifestyle characteristics, including sex, marital status, education level, sleep duration, smoking status, and drinking status; (4) missing data on urban/rural residence, cooking fuel, household consumption, or physical activity; (5) missing data on clinical conditions, including hypertension, diabetes, and hyperlipidemia; (6) missing key laboratory or anthropometric indicators; and (7) missing air pollution exposure data. This left 7420 participants eligible for the baseline cross-sectional analysis. For the longitudinal analysis, we re-extracted the 2011 cohort using the same criteria except the laboratory indicators, then further excluded participants with pre-existing CVD at baseline, those lost to follow-up, and those with missing CVD outcome at follow-up, resulting in a final cohort of 8792 participants (2008 incident CVD events; Figure S1).

### 2.3. Air Pollutant Assessment

Air pollution data, including PM_2.5_, PM_10_, NO_2_, and O_3_, were obtained from the ChinaHighAirPollutants (CHAP) dataset (https://weijing-rs.github.io/product.html, accessed on 11 July 2025). The data were generated through spatiotemporal extreme random forest models, which integrated a range of predictive features including meteorological variables, land use data, pollutant emissions, population distribution, and other spatial and temporal predictors. The CHAP dataset leverages multi-source satellite remote sensing and artificial intelligence technologies to account for the spatiotemporal heterogeneity of air pollution. It derives high-resolution pollutant concentrations by integrating extensive ground-based observations with satellite remote sensing products, atmospheric reanalysis data, and model simulations [14]. Due to confidentiality requirements in the CHARLS database, participant residences were geocoded only to the city level. Consequently, we assigned each participant the city-wide average air pollution concentration, meaning all individuals residing in the same city shared an identical exposure value. Exposure for each survey wave was defined as the mean pollutant concentration over the year preceding the participant’s interview date [15]. Long-term exposure was defined as the 2010–2019 city-level annual mean concentration of each pollutant, assigned to each participant by city.

### 2.4. Revised Definition of Study Outcomes

Heart disease was the primary outcome, and stroke was a secondary outcome. Heart disease was defined based on self-reported physician diagnoses in response to the question: “Have you been diagnosed with heart attack, coronary heart disease, angina, congestive heart failure, or other heart problems by a doctor?” Stroke was defined similarly with the question: “Have you been diagnosed with stroke by a doctor?”

### 2.5. Assessment of Covariates

Demographic characteristics, lifestyle behaviors, and health status were assessed using a standardized, self-administered questionnaire. Potential confounders included demographic factors (age, sex, marital status, education), lifestyle factors (sleep duration, smoking, drinking, moderate-to-vigorous physical activity), anthropometric and clinical measures (BMI, waist circumference, systolic and diastolic blood pressure), and socioeconomic and environmental factors (urban/rural residence, clean cooking fuel, household per capita consumption). Hypertension, diabetes, and hyperlipidemia were based on self-reported diagnoses. Participants were asked: “Have you ever been diagnosed by a doctor with hypertension?”, “Have you ever been diagnosed by a doctor with diabetes?”, and “Have you ever been diagnosed by a doctor with hyperlipidemia?” The TyG index was calculated as Ln [fasting triglycerides (mg/dL) × fasting glucose (mg/dL)/2]. Baseline measurements of fasting blood glucose and triglycerides were obtained from venous blood samples. Anthropometric data, including weight, height, and WC, were collected by trained staff at mobile examination centers using standardized protocols. BMI was calculated as weight divided by height squared (kg/m^2^). The TyG-based indices were derived as follows: TyG-WC = TyG × WC; TyG-BMI = TyG × BMI. Hypertension, diabetes, and dyslipidemia, as well as the TyG, TyG-BMI, and TyG-WC indices, were treated as cardiometabolic outcomes (potential mediators) rather than confounders, and were therefore analyzed as separate outcomes rather than adjusted in the main CVD models.

### 2.6. Statistical Analysis

All data processing and analyses were conducted in R (4.6.1) and Python (3.13.9). The normality of all continuous variables was evaluated using the Kolmogorov–Smirnov test. The results indicated a significant deviation from a normal distribution for all variables (Table S1, all *p*-values < 0.001). Therefore, all continuous variables were presented as median and interquartile range (IQR). Categorical variables were expressed as frequencies and percentages (*n*, %). For comparisons between two groups, the Mann–Whitney U test was employed for continuous variables due to their non-normal distribution. To evaluate potential selection bias from the complete-case analysis, we compared the included (*n* = 7420) and excluded (*n* = 10,288) participants. Correlation analyses were conducted using Spearman’s rank correlation coefficient. A two-sided alpha level of 0.05 was used to determine statistical significance. To account for multiple testing across the primary association analyses, we applied the Benjamini–Hochberg false discovery rate (FDR) correction to the cross-sectional pollutant–outcome comparisons and to the four primary longitudinal comparisons; associations with an FDR-adjusted *q* value < 0.05 were considered statistically significant.

In the cross-sectional analysis, we used logistic regression and Spearman’s correlation to assess the associations of air pollutant levels with CVD and its established risk factors. As a sensitivity analysis to assess the robustness of the complete-case results, we performed multiple imputation by chained equations (MICE, m = 20) using predictive mean matching. Baseline covariates with missing values (demographic, clinical, laboratory, and additional covariates) were imputed, and the derived variables (CVD, the TyG-related indices, and physical activity) were recomputed from the imputed values (“impute raw, derive later”). Estimates were pooled using Rubin’s rules and compared with the complete-case estimates. Furthermore, we evaluated the associations between air pollution and metabolic indicators (TyG, TyG-BMI, TyG-WC).

We used follow-up data from waves 2–5 (2013–2020) to ascertain incident CVD. In the longitudinal analysis, we used Cox proportional hazards regression, with the long-term (2010–2019 mean) exposure to each pollutant as the main exposure variable. We assessed the proportional hazards assumption using Schoenfeld residuals and used cluster-robust standard errors (clustered by city) to account for within-city correlation. We performed subgroup analyses by urban/rural residence as a sensitivity analysis. To assess the independent associations given the correlation among pollutants, we additionally fitted two-pollutant models (each pair of pollutants entered simultaneously).

To evaluate the predictive value of air pollutants and baseline characteristics for cardiovascular risk, we built a random forest (RF) model in the longitudinal cohort (*n* = 8792 participants free of CVD at baseline; 2008 incident CVD events over follow-up). The model included 22 baseline predictors: long-term exposure to PM_2.5_, PM_10_, NO_2_, and O_3_ (2010–2019 city-level annual mean concentrations); demographic and lifestyle factors (age, sex, marital status, education, sleep duration, smoking, drinking); clinical characteristics (systolic and diastolic blood pressure, BMI, waist circumference, hypertension, diabetes, dyslipidemia); and socioeconomic and environmental factors (urban/rural residence, clean cooking fuel, household per capita consumption, moderate-to-vigorous physical activity). Model performance was assessed by repeated 5-fold cross-validation (10 repeats). We report the area under the receiver operating characteristic curve (AUC) with its 95% confidence interval (CI), together with sensitivity, specificity, positive predictive value (PPV), negative predictive value (NPV), and the Brier score for calibration. Variable importance was estimated by permutation importance (% increase in out-of-bag error), which is less biased than Gini importance when predictors are correlated. Analyses were performed in R using the ranger package.

## 3. Results

### 3.1. Air Pollution Increases the Burden of CVD

**Figure 1:**
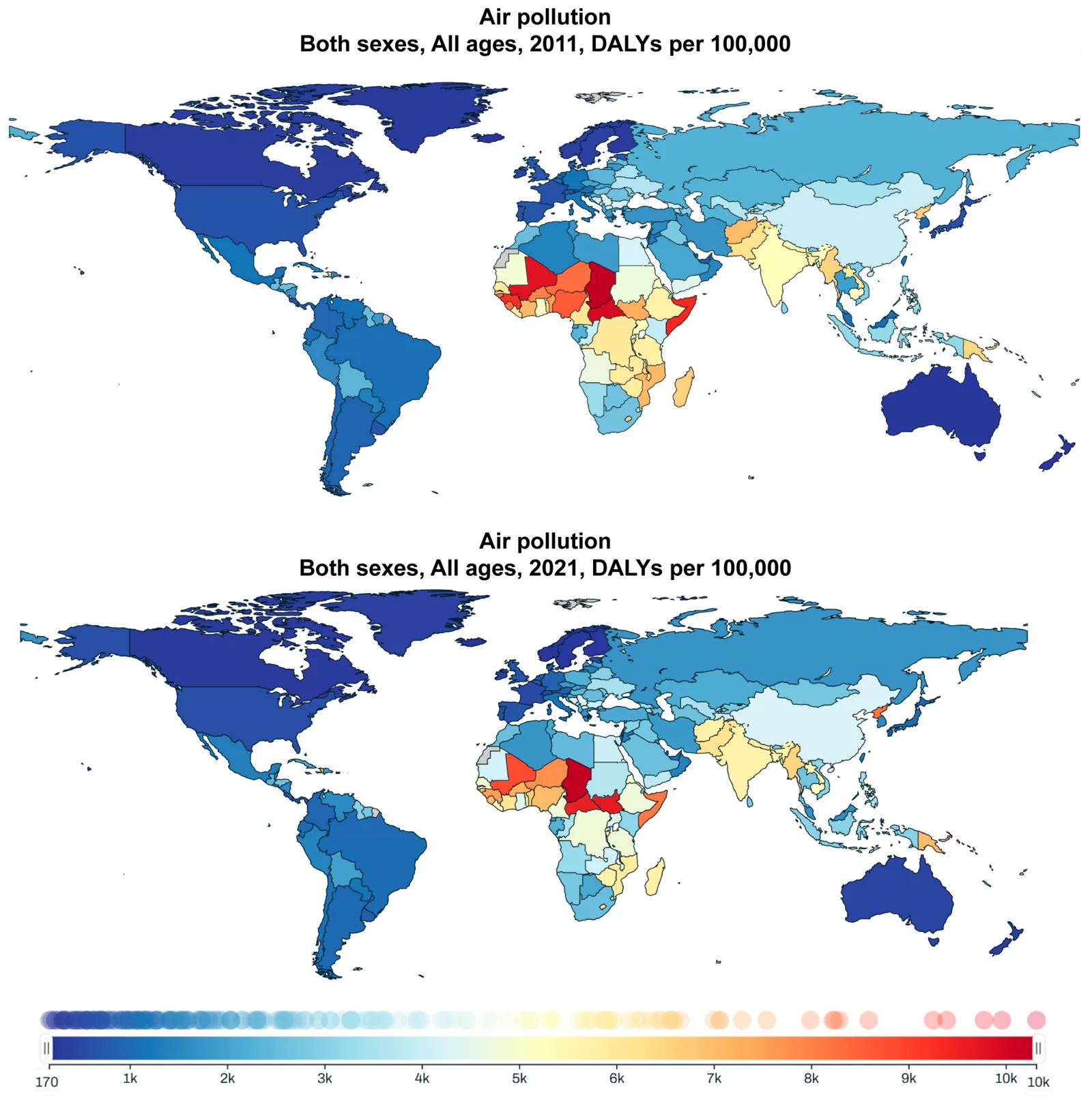
Global disease burden attributable to air pollution. The disease burden was measured in Disability-Adjusted Life Years (DALYs). Color intensity corresponds to the magnitude of the burden, with red indicating higher levels and blue indicating lower levels.

**Figure 2:**
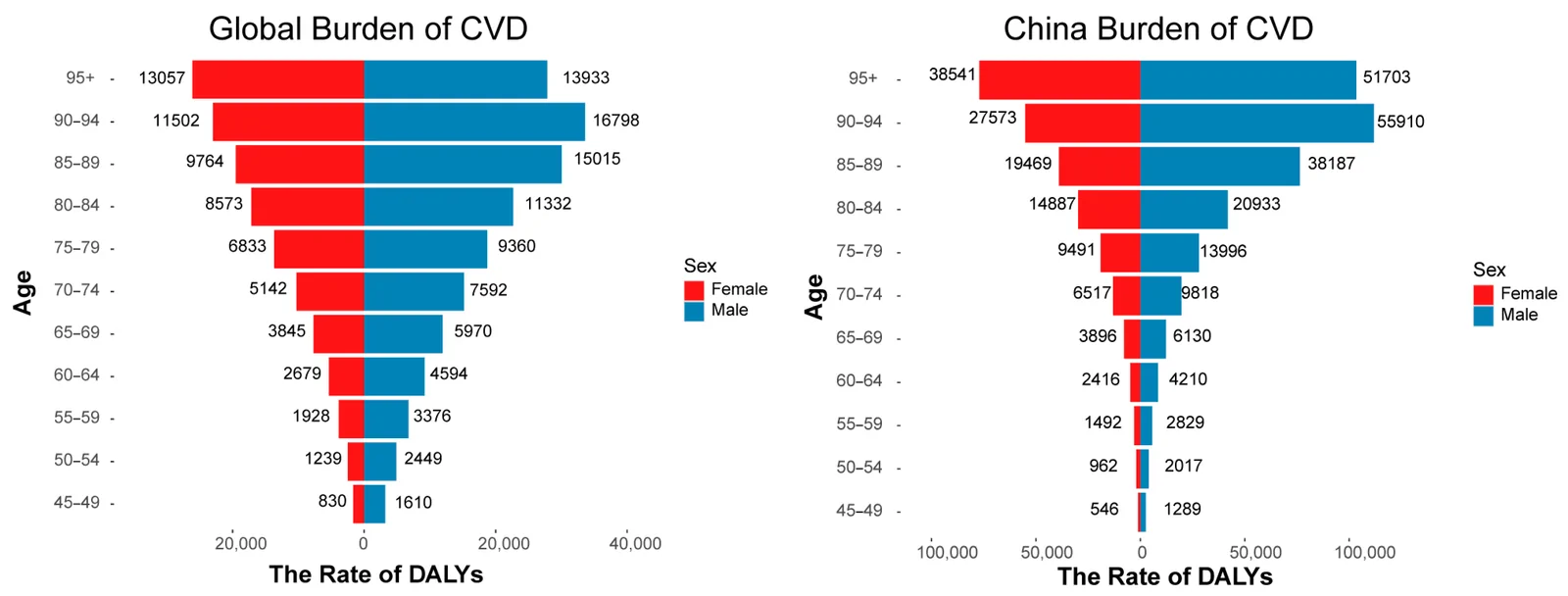
CVD burden from air pollution: mortality and DALYs per 100,000 population (Global and China, 2021). CVD, cardiovascular disease. DALYs, disability-adjusted life years.

The updated GBD 2021 database revealed that over the past decade, air pollution levels have undergone modest changes (Figure 1). While highly polluted regions remain concentrated in Africa, their pollution levels in 2021 show a slight decline compared to those in 2011. China’s air pollution-attributable DALYs per 100,000 population decreased from 3988.8 (95% UI: 3389.33–4621.25) in 2011 to 2452.03 (95% UI: 1823.97–3128.84) in 2021 (Figure 1). However, further analysis on the burden of air pollution-attributable CVD among middle-aged and older adults (aged ≥ 45 years) revealed that this subgroup maintained a persistently high burden in 2021 (Figure 2). This burden demonstrated a clear age-related increasing trend, which was more pronounced in China than the global average. Notably, among individuals aged 80 years and above, the attributable DALY rate exceeded 10,000 per 100,000 population, with a higher burden observed in males compared to females (Figure 2).

### 3.2. Baseline Characteristics

A total of 7420 participants were finally included: 6390 without CVD and 1030 with CVD (Table 1). The cohort’s mean age was 58.5 years, with a gender distribution of 47.2% male participants. Educational attainment was predominantly high school level or lower, and the majority of participants were married. Significant differences between the two groups were observed in age, gender, and multiple clinical and lifestyle metrics, including sleep duration, drinking, smoking, and blood pressure. PM_10_ and PM_2.5_ exposure levels showed a statistically significant difference between groups stratified by CVD status. As shown in Supplementary Table S2, the two groups were largely comparable in terms of CVD prevalence (13.9% vs. 14.7%, *p* = 0.150), as well as other major comorbidities, suggesting that data were unlikely to be missing in direct relation to the outcome.

### 3.3. Cross-Sectional Associations of Pollutants with CVD

**Figure 3:**
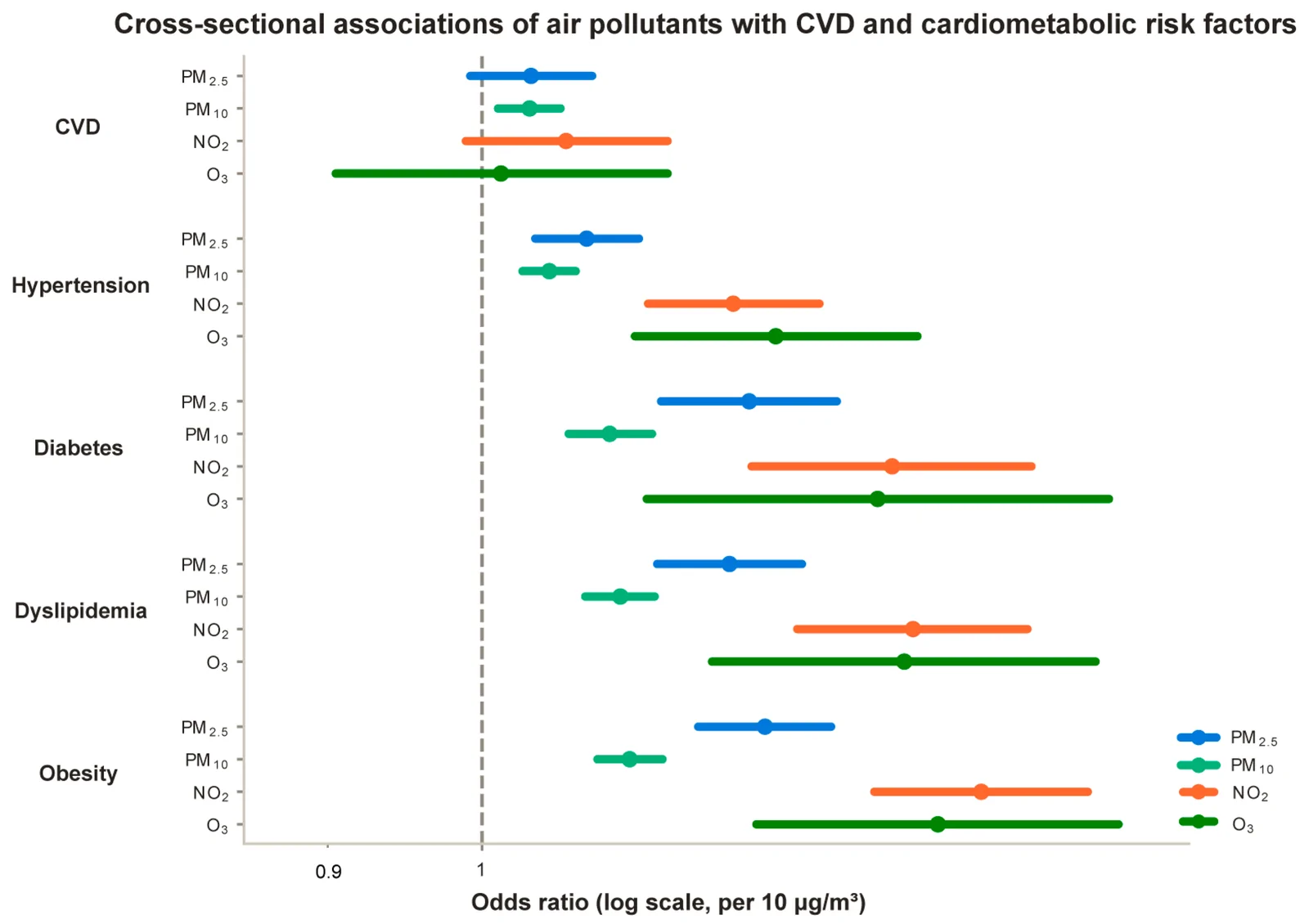
Forest plot of cross-sectional associations between air pollutants and cardiometabolic outcomes from logistic regression models.

To evaluate the association between pollutants and CVD, we conducted logistic regression using 2011 baseline data from 7420 participants to estimate ORs for each pollutant (Figure 3). The models were adjusted for the following baseline covariates: age, sex, marital status, education level, systolic and diastolic blood pressure, sleep duration, smoking and drinking history, urban/rural residence, clean cooking fuel, household per capita consumption, and moderate-to-vigorous physical activity. The results of the cross-sectional logistic regression analysis indicated that each 10 μg/m^3^ increase in PM_10_ exposure was associated with a significantly increased risk of CVD (OR: 1.033, 95% CI: 1.011–1.055). In contrast, increases of 10 μg/m^3^ in exposure to PM_2.5_ (OR: 1.034, 95% CI: 0.992–1.078), NO_2_ (OR: 1.059, 95% CI: 0.989–1.135), or O_3_ (OR: 1.013, 95% CI: 0.905–1.135) were not significantly associated with CVD risk. However, all pollutants were significantly associated with cardiometabolic risk factors (hypertension, diabetes, dyslipidemia, and obesity). These associations were strongest for NO_2_ exposure, with each 10 μg/m^3^ increase being linked to a higher risk of hypertension (OR: 1.187; 95% CI: 1.120–1.259), diabetes (OR: 1.323; 95% CI: 1.202–1.455), dyslipidemia (OR: 1.342; 95% CI: 1.240–1.451), and obesity (OR: 1.406; 95% CI: 1.307–1.512). All of these pollutant–cardiometabolic associations, as well as the PM_10_–CVD association, remained statistically significant after Benjamini–Hochberg FDR correction (all q < 0.05, Table S3).

To examine whether the complete-case analysis was subject to selection bias, we performed multiple imputation (MICE, m = 20) that restored age-eligible participants with missing covariates to the analysis (*n* = 17,060, Figure S2). The resulting ORs were closely aligned with those from the complete-case analysis (*n* = 7420): PM_10_, 1.025 (95% CI: 1.011–1.039) vs. 1.033 (95% CI: 1.011–1.055); PM_2.5_, 1.011 (95% CI: 0.984–1.039) vs. 1.034 (95% CI: 0.992–1.078); NO_2_, 1.008 (95% CI: 0.962–1.055) vs. 1.059 (95% CI: 0.989–1.135); and O_3_, 0.920 (95% CI: 0.853–0.992) vs. 1.013 (95% CI: 0.905–1.135). All confidence intervals across the two methods showed substantial overlap, indicating that the complete-case estimates were robust to potential selection bias due to missing data.

Spearman’s correlation analysis also revealed significant associations between air pollutant levels and key metabolic indicators, including TyG, TyG-BMI, and TyG-WC (Figure S3). Moreover, PM_2.5_, PM_10_, NO_2_, and O_3_ exhibited significantly stronger correlations with TyG-BMI and TyG-WC than with TyG.

### 3.4. Longitudinal Associations of Pollutants with CVD

**Figure 4:**
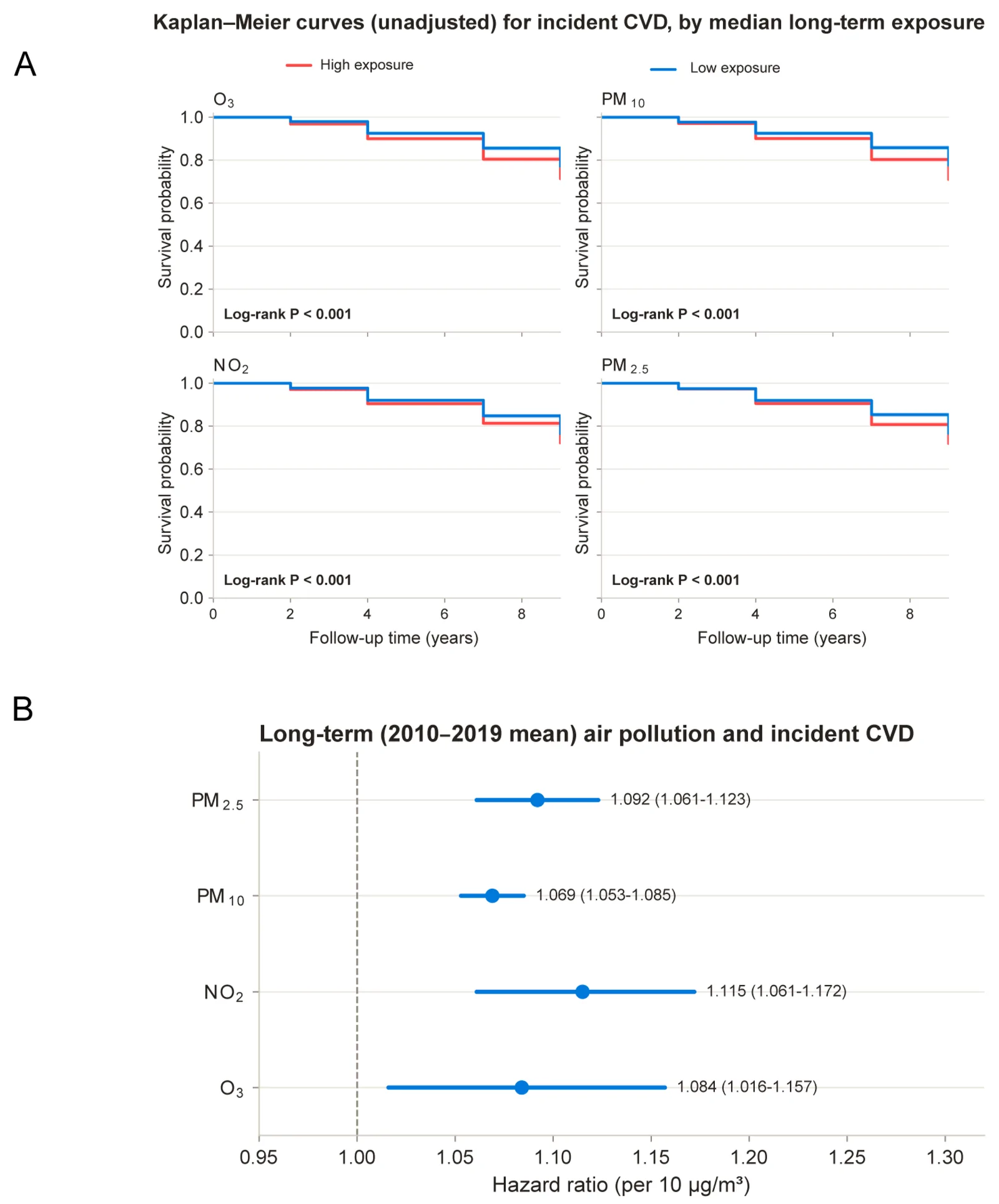
Association between long-term exposure to PM_2.5_, PM_10_, NO_2_, and O_3_ and incident CVD. (**A**) Kaplan–Meier survival curves stratified by high versus low exposure (median split), with log-rank P values. (**B**) Hazard ratios per 10 µg/m^3^ increase from Cox proportional hazards models with cluster-robust standard errors (clustered by city).

During a mean follow-up of 7.57 years, 2008 of the 8792 participants free of CVD at baseline (22.8%) developed incident CVD. Kaplan–Meier (unadjusted) survival curves, stratified by the median of the long-term (2010–2019 mean) exposure concentration, showed that participants in the high-exposure group had a significantly lower survival probability than those in the low-exposure group for all four pollutants (log-rank *p* < 0.001 for PM_2.5_, PM_10_, NO_2_, and O_3_; Figure 4).

In the primary Cox proportional hazards model with cluster-robust standard errors (clustered by city, Figure S4A), each 10 μg/m^3^ increase in long-term exposure was significantly associated with an increased risk of incident CVD for PM_2.5_ (HR = 1.09, 95% CI: 1.03–1.16; *p* = 0.005) and PM_10_ (HR = 1.07, 95% CI: 1.04–1.10; *p* < 0.001). In contrast, the association for NO_2_ was marginal (HR = 1.12, 95% CI: 1.00–1.24; *p* = 0.047), and that for O_3_ was not statistically significant (HR = 1.08, 95% CI: 0.93–1.27; *p* = 0.308). After Benjamini–Hochberg FDR correction across the four pollutants, PM_2.5_ (q = 0.009) and PM_10_ (q < 0.001) remained significantly associated with incident CVD, whereas NO_2_ (q = 0.062) and O_3_ (q = 0.308) did not (Table S3). The proportional hazards assumption was assessed using Schoenfeld residuals (Figure S4B); minor deviations were observed for some pollutants (global *p* = 0.015–0.071), and the hazard ratios are therefore interpreted as average effects over follow-up. Subgroup analyses by urban/rural residence showed no significant effect modification (all *p* for interaction > 0.05, Figure S4C). In two-pollutant models, the associations for PM_10_ and PM_2.5_ remained robust, whereas NO_2_ and O_3_ were attenuated after mutual adjustment (Figure S4D).

### 3.5. Random Forest-Based Prediction of Incident CVD

**Figure 5:**
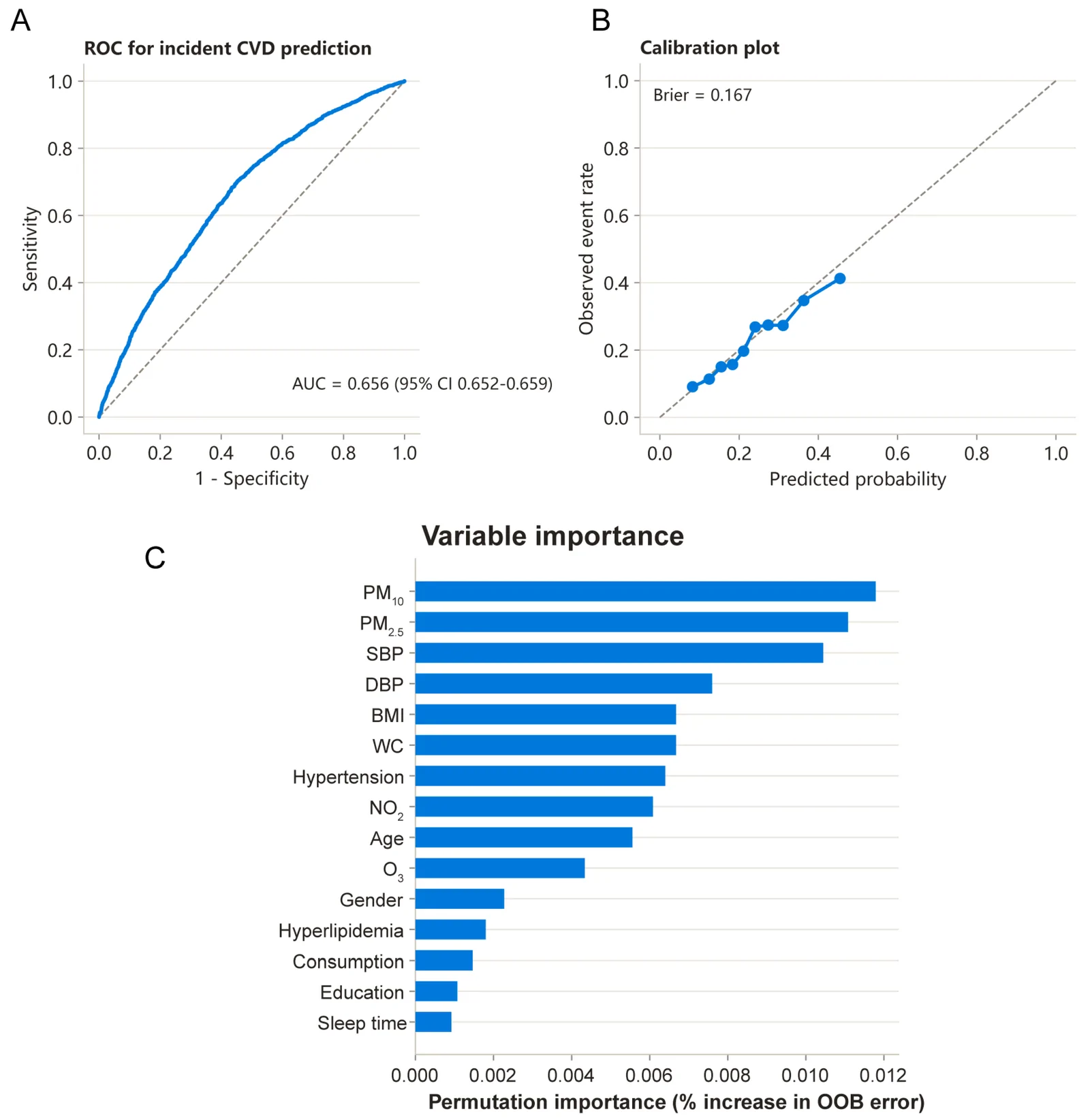
Random forest prediction of incident cardiovascular disease. (**A**) Receiver operating characteristic curve (repeated 5-fold cross-validation; AUC = 0.656, 95% CI 0.652–0.659). (**B**) Calibration plot (Brier score = 0.167). (**C**) Permutation variable importance (% increase in out-of-bag error). The model included long-term air pollutant exposure, demographic, lifestyle, clinical, and socioeconomic predictors.

The random forest model showed modest discrimination for incident CVD, with an AUC of 0.656 (95% CI 0.652–0.659) in repeated 5-fold cross-validation (Figure 5A). At the optimal (Youden) threshold, sensitivity was 0.703, specificity 0.546, positive predictive value 0.314, and negative predictive value 0.861; the Brier score was 0.167, indicating acceptable calibration (Figure 5B). Permutation importance identified PM_10_ and PM_2.5_ as the two most important predictors, followed by systolic and diastolic blood pressure, BMI, waist circumference, and hypertension; NO_2_ and O_3_ also ranked among the top ten (Figure 5C). Thus, ambient air pollutants were among the leading contributors to incident CVD prediction in this model, whereas the additional socioeconomic and environmental covariates (urban/rural residence, cooking fuel, household consumption, physical activity) contributed little to predictive performance.

## 4. Discussion

This study provided a comprehensive analysis of the air pollution-associated CVD burden in adults aged ≥ 45 years. By leveraging multi-source data from the GBD, CHAP, and CHARLS studies, it combined cross-sectional and longitudinal analyses to demonstrate that air pollution, particularly PM_10_, increases the risk of CVD and metabolic disorders. We also developed a random forest model incorporating long-term air pollutant exposure and baseline clinical, lifestyle, and socioeconomic characteristics to predict incident CVD, which showed modest discrimination (AUC = 0.656, 95% CI 0.652–0.659).

Air pollution has emerged as a critical environmental health crisis with profound implications for global public health. As our results showed, older age corresponded to higher rates of DALYs attributable to air pollution, indicating a greater overall disease burden. In the prospective analysis, long-term exposure to PM_2.5_ and PM_10_ was significantly associated with an increased risk of incident CVD, whereas the associations for NO_2_ and O_3_ were weaker and not robust to clustering. Notably, exposure to each of these pollutants was independently associated with an increased risk of hypertension. Mounting evidence also links air pollution to an increased risk of CVD [16,17,18]. Long-term exposure to PM_2.5_ was associated with an elevated relative risk of first-time hospitalization due to various CVDs—including ischemic heart disease, heart failure, cardiomyopathy, arrhythmia, cerebrovascular disease, and thoracic and abdominal aortic aneurysms—among adults aged 65 and older in the United States [19]. A significant association was also observed between long-term PM_2.5_ exposure and increased CVD risk in a large-scale cohort of 116,972 Chinese adults [20]. Each 10 μg/m^3^ increase in the 7-day moving average PM_10_ concentration was significantly associated with a 2.48% increase in the risk of hospitalization for CVD [21]. A study conducted in a Chinese adult population found that increased exposure to NO_2_ was significantly associated with elevated risks of overall CVD, hypertension, and stroke [22]. Current evidence regarding the association between O_3_ exposure and CVD remains controversial, with some studies suggesting an increased risk [23,24,25], while others—consistent with the findings of this study—report no significant association [26,27,28]. These discrepancies may be attributable to regional heterogeneity, variations in exposure assessment accuracy, differences in population characteristics, and diversity in O_3_ exposure concentrations. Consequently, additional well-designed cohort studies are warranted to better characterize the cardiovascular effects of long-term O_3_ exposure.

Cardiac metabolic dysfunction plays a critical role in the pathogenesis and progression of CVD. It is well established that dysregulation of glucose and lipid metabolism, encompassing both hyperglycemic states and lipid metabolic aberrations, exerts deleterious effects on cardiac structure and function [29]. Our investigation also corroborated that these air pollutants confer detrimental impacts on cardiometabolic risk factors, including obesity, diabetes mellitus, hyperlipidemia, and hypertension, as evidenced by their elevated correlation coefficients with TyG, TyG-BMI, and TyG-WC. We employed a random forest model incorporating air pollutants and baseline characteristics to predict incident CVD; permutation importance identified PM_2.5_ and PM_10_ as the two most important predictors, indicating that air pollution contributes meaningfully to cardiovascular risk prediction. Studies have reported that exposure to air pollution is associated with these cardiometabolic risk factors [30,31]. Exposure to air pollution is significantly associated with an increased risk of obesity in both children and adults [32,33,34]. A large-scale UK cohort study with nearly 400,000 participants and a follow-up period of 12 years revealed that exposure to PM_10_, PM_2.5_, and NO_2_ was associated with an elevated risk of developing type 2 diabetes mellitus (T2DM) and its related complications [35]. Long-term exposure to low-concentration O_3_ is also significantly associated with a higher incidence of T2DM [36]. Exposure to air pollution is also associated with an elevated risk of hyperlipidemia [31,37] and hypertension [31,38].

Oxidative stress represents a central mechanism mediating the adverse effects of air pollution on cardiovascular health [39]. Upon inhalation, air pollution first induces oxidative stress primarily within the airways and lungs. Exposure to atmospheric particulate matter triggers an inflammatory response in pulmonary epithelial cells and alveolar macrophages, and these particles may exert systemic pro-inflammatory and pro-oxidative effects once they enter the bloodstream [40]. Following intratracheal instillation of particulate matter in rats, a marked elevation in cardiac oxidative stress levels was observed [41]. Scavenging free radicals effectively mitigates the direct cardiotoxic effects induced by diesel exhaust particles in neonatal rat cardiomyocytes [42].

This study comprehensively examined the association between exposure to multiple ambient air pollutants (PM_2.5_, PM_10_, O_3_, and NO_2_) and the incidence of CVD using nationwide data from adults aged ≥ 45 years. By combining cross-sectional and cohort designs, this study provides evidence that lends support to a potential causal relationship. This study further identified significant associations between these air pollutants and various cardiometabolic risk factors. Several limitations of this study should be considered. First, our assessment was limited by the coarse spatial granularity of the air pollution data, which was available only at the city level and precluded analysis of finer-scale variations. Second, disease diagnoses were based on self-reported information, which is subject to potential misclassification bias due to the inclusion of undiagnosed cases. Third, due to data unavailability, we were unable to analyze or control for the influence of additional gaseous pollutants, such as sulfur dioxide (SO_2_) or carbon monoxide (CO). Fourth, even after extensive adjustment for known confounders, confounding from unmeasured variables cannot be excluded.

## 5. Conclusions

This study integrated data from the GBD, CHAP, and CHARLS sources to evaluate the cardiovascular burden of air pollution in middle-aged and older Chinese adults. Long-term exposure to PM_2.5_ (HR = 1.09, 95% CI: 1.03–1.16) and PM_10_ (HR = 1.07, 95% CI: 1.04–1.10) was significantly associated with an elevated risk of incident cardiovascular disease, whereas the associations for NO_2_ and O_3_ were weaker and not robust to clustering by city. All four pollutants were additionally associated with adverse cardiometabolic profiles—including hypertension, diabetes, dyslipidemia, and obesity—and with higher TyG-related indices. A random forest model identified PM_10_ and PM_2.5_ as the two most important predictors of incident CVD, underscoring the contribution of particulate matter to cardiovascular risk beyond conventional risk factors. Taken together, these findings support sustained reductions in ambient particulate matter as an important public-health strategy to alleviate the growing cardiovascular burden in aging populations.

## Figures and Tables

**Table 1:** Baseline characteristics of the population stratified by CVD.

	Total (7420)	No CVD (6390)	CVD (1030)	*p* Value
Age (years), M (Q1, Q3)	58.5 [52.0, 65.0]	58.0 [52.0, 65.0]	61.0 [55.0, 68.0]	**<0.001**
Male, *n* (%)	3505 (47.2)	3067 (48.0)	438 (42.5)	**0.001**
Married, *n* (%)	6311 (85.1)	5454 (85.4)	857 (83.2)	0.081
Secondary education and below, *n* (%)	6730 (90.7)	5808 (90.9)	922 (89.5)	0.175
Rural, *n* (%)	6202 (83.6)	5433 (85.0)	769 (74.7)	**<0.001**
Sleep time (hours), M (Q1, Q3)	6.0 [5.0, 8.0]	6.5 [5.0, 8.0]	6.0 [5.0, 7.0]	**<0.001**
Drinking, *n* (%)	2441 (32.9)	2188 (34.2)	253 (24.6)	**<0.001**
Smoking, *n* (%)	2290 (30.9)	2027 (31.7)	263 (25.5)	**<0.001**
Moderate-to-vigorous physical activity, *n* (%)	3328 (44.9)	2853 (44.6)	475 (46.1)	0.398
Clean cooking fuel, *n* (%)	4400 (59.3)	3791 (59.3)	609 (59.1)	0.930
Household consumption per capita (yuan), M (Q1, Q3)	4495.3 [2632.6, 7727.1]	4433.8 [2613.6, 7623.8]	4840.2 [2743.4, 8386.4]	**0.002**
SBP (mmHg), M (Q1, Q3)	126.5 [113.5, 141.5]	126.0 [113.0, 140.5]	130.5 [116.1, 146.4]	**<0.001**
DBP (mmHg), M (Q1, Q3)	74.5 [66.5, 83.0]	74.0 [66.5, 82.5]	76.0 [68.0, 85.0]	**<0.001**
BMI, M (Q1, Q3)	23.1 [20.9, 25.8]	23.0 [20.8, 25.6]	24.0 [21.4, 26.9]	**<0.001**
Waist (cm), M (Q1, Q3)	84.6 [77.9, 92.0]	84.1 [77.4, 91.4]	87.0 [79.6, 95.0]	**<0.001**
Hypertension, *n* (%)	1946 (26.2)	1431 (22.4)	515 (50.0)	**<0.001**
Diabetes, *n* (%)	462 (6.2)	331 (5.2)	131 (12.7)	**<0.001**
Dyslipidemia, *n* (%)	712 (9.6)	470 (7.4)	242 (23.5)	**<0.001**
CRP (mg/L), M (Q1, Q3)	1.0 [0.6, 2.2]	1.0 [0.5, 2.1]	1.2 [0.7, 2.6]	**<0.001**
HbA1c (%), M (Q1, Q3)	5.1 [4.9, 5.4]	5.1 [4.9, 5.4]	5.2 [4.9, 5.5]	**<0.001**
Hemoglobin (g/L), M (Q1, Q3)	14.2 [13.0, 15.5]	14.2 [13.0, 15.5]	14.3 [13.2, 15.6]	**0.047**
Total cholesterol (mg/dL), M (Q1, Q3)	190.6 [167.4, 215.3]	190.2 [167.4, 215.3]	192.5 [169.0, 217.3]	0.075
Triglycerides (mg/dL), M (Q1, Q3)	105.3 [75.2, 154.0]	103.5 [74.3, 150.4]	118.6 [84.1, 176.1]	**<0.001**
HDL-C (mg/dL), M (Q1, Q3)	49.5 [40.2, 59.9]	49.9 [40.6, 60.3]	46.0 [38.3, 57.6]	**<0.001**
LDL-C (mg/dL), M (Q1, Q3)	114.4 [93.2, 137.2]	114.0 [93.2, 136.9]	116.2 [93.9, 138.3]	0.147
Glucose (mg/dL), M (Q1, Q3)	102.6 [94.5, 113.8]	102.2 [94.3, 113.0]	104.4 [96.0, 118.8]	**<0.001**
TyG, M (Q1, Q3)	8.6 [8.2, 9.1]	8.6 [8.2, 9.0]	8.8 [8.4, 9.2]	**<0.001**
TyG-WC, M (Q1, Q3)	728.8 [651.9, 817.8]	722.8 [648.5, 812.0]	765.9 [673.7, 857.1]	**<0.001**
TyG-BMI, M (Q1, Q3)	199.8 [175.7, 229.7]	198.2 [174.9, 227.1]	211.5 [182.3, 242.7]	**<0.001**
PM_2.5_ (μg/m^3^), M (Q1, Q3)	56.1 [42.3, 67.3]	56.1 [42.2, 67.3]	56.1 [44.0, 69.3]	**0.049**
PM_10_ (μg/m^3^), M (Q1, Q3)	96.7 [70.1, 115.7]	96.7 [68.3, 113.4]	96.8 [76.5, 119.8]	**0.002**
NO_2_ (μg/m^3^), M (Q1, Q3)	28.2 [21.8, 36.9]	28.2 [21.8, 36.9]	28.2 [22.3, 37.3]	0.344
O_3_ (μg/m^3^), M (Q1, Q3)	83.1 [79.5, 88.0]	83.1 [79.5, 88.0]	83.3 [79.5, 88.0]	0.344

Continuous variables were presented as median and interquartile range. Categorical variables were expressed as frequencies and percentages (*n*, %). CVD, cardiovascular disease; SBP, systolic blood pressure; DBP, diastolic blood pressure; HDL-C, high-density lipoprotein cholesterol; LDL-C, low-density lipoprotein cholesterol; Hb, hemoglobin; CRP, C-reactive protein; TyG, Triglyceride-Glucose Index; WC, waist circumference; BMI, body mass index; Bold indicates statistical significance.

## Data Availability

The data for this retrospective study were sourced from the CHARLS database. Data are contained within the article and Supplementary Materials, and no ethical issues were identified. The ethical approval for the CHARLS project was granted by the Ethical Review Committee of Peking University (IRB00001052-11015), and all participants signed informed consent forms during the initial enrollment. This study complies with the Declaration of Helsinki.

## Supplementary Materials

The following supporting information can be downloaded at: https://www.mdpi.com/article/10.3390/bioengineering13090958/s1, Figure S1: Flowchart of the sample selection process; Table S1: Normality test results for the study variables; Table S2: Comparison of baseline characteristics between included and excluded participants; Figure S2: Comparison of cross-sectional associations between air pollutants and CVD risk between complete-case analysis and multiple imputation sensitivity analysis; Table S3: Multiple-testing correction (Benjamini–Hochberg FDR) for pollutant associations with cardiovascular and cardiometabolic outcomes; Figure S3: Spearman correlation analysis of air pollutant levels and key metabolic indicators; Figure S4. Association between long-term exposure to PM_2.5_, PM_10_, NO_2_, and O_3_ and incident CVD.
