# Supplementary material for: Long-Term Air Pollution Exposure, Cardiovascular Disease, and Cardiometabolic Risk Factors in Middle-Aged and Older Chinese Adults: A Prospective Cohort Study
Source: Bioengineering (Basel). 2026 Aug 23;13(9):958. doi: 10.3390/bioengineering13090958 (PMC13603370; doi:10.3390/bioengineering13090958)
Supplement: Supplementary file 1 [file bioengineering-13-00958-s001.zip › bioengineering-4513100-supplementary.pdf]

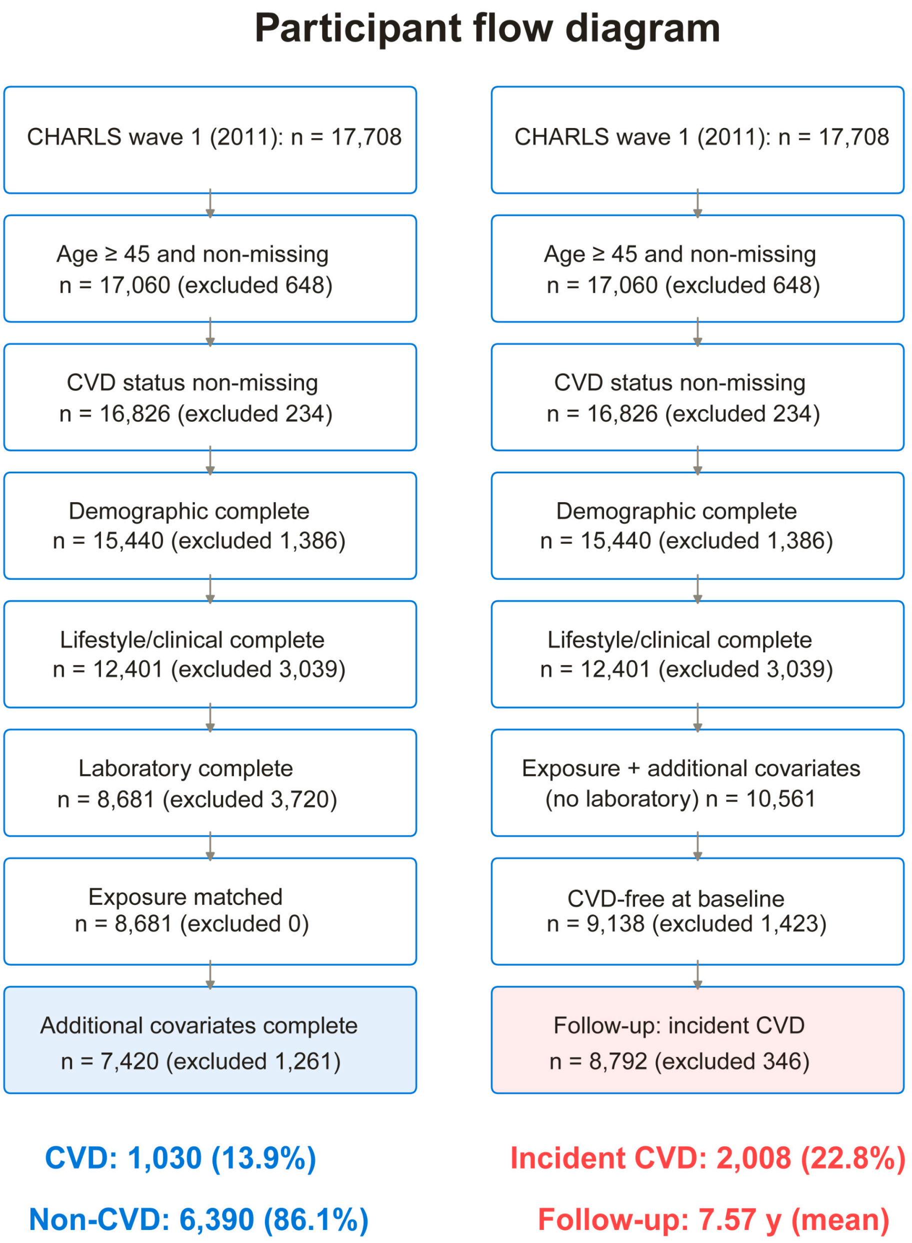

Figure S1. Flowchart of the sample selection process.

Table S1 Normality test results for the study variables

	KS_p.value	Normality_KS
Sleep (hours)	**<0.001**	Non-normal
SBP (mmHg)	**<0.001**	Non-normal
DBP (mmHg)	**<0.001**	Non-normal
BMI	**<0.001**	Non-normal
Waist (cm)	**<0.001**	Non-normal
Household consumption per capita (yuan)	**<0.001**	Non-normal
CRP (mg/l)	**<0.001**	Non-normal
HbA1c (%)	**<0.001**	Non-normal
Hb (g/l)	**<0.001**	Non-normal
Total Cholesterol (mg/dl)	**<0.001**	Non-normal
Triglycerides (mg/dl)	**<0.001**	Non-normal
HDL-C(mg/dl)	**<0.001**	Non-normal
LDL-C(mg/dl)	**<0.001**	Non-normal
Glucose (mg/dl)	**<0.001**	Non-normal
Age (years)	**<0.001**	Non-normal
$PM_{2.5}$ (μg/m^3)	**<0.001**	Non-normal
O_3 (μg/m^3)	**<0.001**	Non-normal
NO_2 (μg/m^3)	**<0.001**	Non-normal
PM_{10} (μg/m^3)	**<0.001**	Non-normal
TyG	**<0.001**	Non-normal
TyG-WC	**<0.001**	Non-normal
TyG-BMI	**<0.001**	Non-normal

SBP, systolic blood pressure; DBP, diastolic pressure; HDL-C, high density lipoprotein cholesterol; LDL-C, low density lipoprotein cholesterol; Hb, hemoglobin; CRP, C-reactive protein; TyG, Triglyceride-Glucose Index; WC, waist circumference; BMI, body mass index.

Table S2 Comparison of baseline characteristics between included and excluded participants

Variable	Included (7420)	Excluded (10288)	P value
Age (years)	58.5 [52.0, 65.0]	57.0 [50.0, 65.0]	**<0.001**
Male	3505 (47.2)	4974 (48.4)	0.145
Married	6311 (85.1)	7859 (76.6)	**<0.001**
Secondary education and below	6730 (90.7)	8795 (85.6)	**<0.001**
Rural	6202 (83.6)	7492 (73.1)	**<0.001**
Sleep time (hours)	6.0 [5.0, 8.0]	7.0 [5.0, 8.0]	**0.008**
Drinking	2441 (32.9)	3326 (32.8)	0.934

Smoking	2290 (30.9)	2581 (27.2)	**<0.001**
Hypertension	1946 (26.2)	2589 (25.8)	0.499
Diabetes	462 (6.2)	600 (6.0)	0.572
Dyslipidemia	712 (9.6)	984 (10.0)	0.337
CVD	1030 (13.9)	1472 (14.7)	0.15
PM2.5 (ug/m3)	56.1 [42.3, 67.3]	50.7 [39.0, 66.3]	**<0.001**
PM10 (ug/m3)	96.7 [70.1, 115.7]	89.1 [63.6, 112.0]	**<0.001**
NO2 (ug/m3)	28.2 [21.8, 36.9]	28.3 [21.7, 36.9]	**0.028**
O3 (ug/m3)	83.1 [79.5, 88.0]	82.6 [79.9, 88.0]	0.344

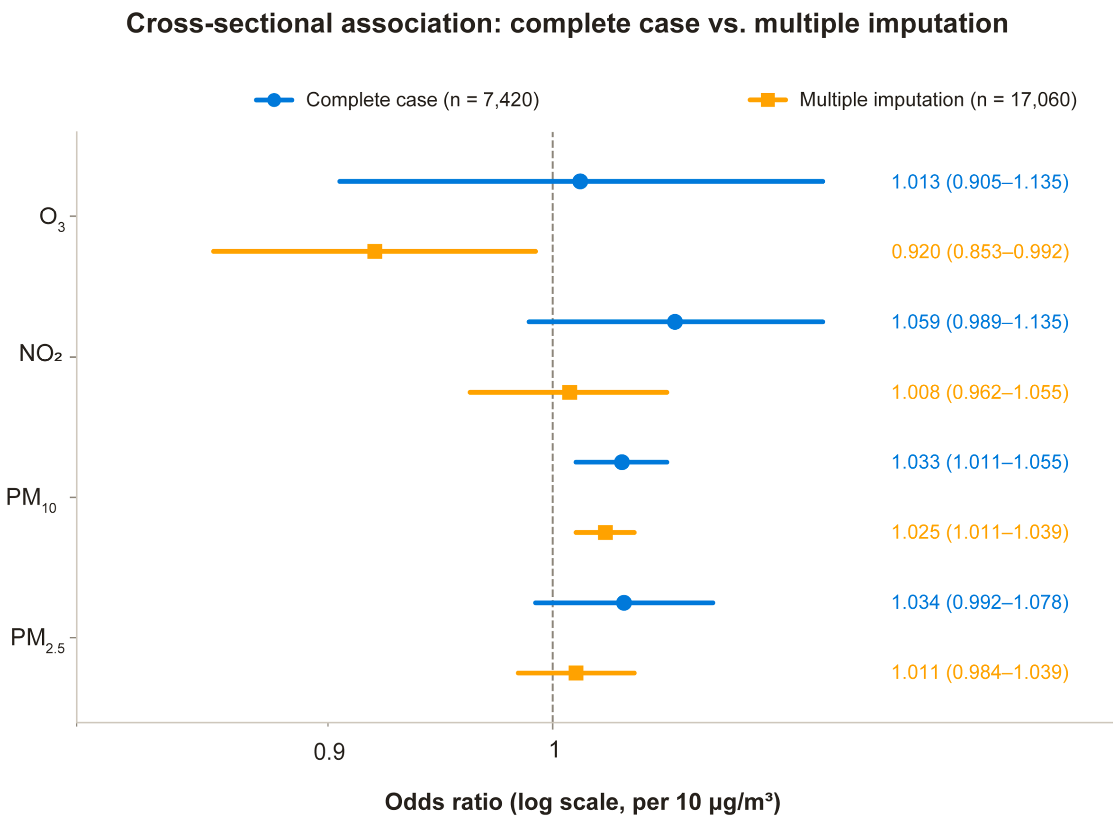

Figure S2. Comparison of cross-sectional associations between air pollutants and CVD risk between complete-case analysis and multiple imputation sensitivity analysis.

Table S3 Multiple-testing correction (Benjamini–Hochberg FDR) for pollutant associations with cardiovascular and cardiometabolic outcomes

Analysis family	Pollutant	Outcome	Effect estimate	P value	q value
Cross-sectional	PM2.5	CVD	1.034166	0.114	0.152
Cross-sectional	PM2.5	Heart Disease	1.049566	**0.032**	**0.047**
Cross-sectional	PM2.5	Stroke	0.972329	0.55	0.67
Cross-sectional	PM2.5	Diabetes	1.200052	**<0.001**	**<0.001**
Cross-sectional	PM2.5	Dyslipidemia	1.18409	**<0.001**	**<0.001**

Cross-sectional	PM2.5	Hypertension	1.073895	**<0.001**	**<0.001**
Cross-sectional	PM2.5	Obesity	1.212592	**<0.001**	**<0.001**
Cross-sectional	PM10	CVD	1.032517	**0.003**	**0.005**
Cross-sectional	PM10	Heart Disease	1.042125	**<0.001**	**<0.001**
Cross-sectional	PM10	Stroke	0.996639	0.889	0.952
Cross-sectional	PM10	Diabetes	1.091468	**<0.001**	**<0.001**
Cross-sectional	PM10	Dyslipidemia	1.098833	**<0.001**	**<0.001**
Cross-sectional	PM10	Hypertension	1.046842	**<0.001**	**<0.001**
Cross-sectional	PM10	Obesity	1.106183	**<0.001**	**<0.001**
Cross-sectional	NO2	CVD	1.059325	0.102	0.143
Cross-sectional	NO2	Heart Disease	1.058478	0.129	0.164
Cross-sectional	NO2	Stroke	1.01929	0.808	0.919
Cross-sectional	NO2	Diabetes	1.322664	**<0.001**	**<0.001**
Cross-sectional	NO2	Dyslipidemia	1.341745	**<0.001**	**<0.001**
Cross-sectional	NO2	Hypertension	1.18749	**<0.001**	**<0.001**
Cross-sectional	NO2	Obesity	1.405773	**<0.001**	**<0.001**
Cross-sectional	O3	CVD	1.013159	0.821	0.919
Cross-sectional	O3	Heart Disease	1.005135	0.934	0.952
Cross-sectional	O3	Stroke	0.992392	0.952	0.952
Cross-sectional	O3	Diabetes	1.310106	**<0.001**	**0.001**
Cross-sectional	O3	Dyslipidemia	1.333719	**<0.001**	**<0.001**
Cross-sectional	O3	Hypertension	1.222399	**<0.001**	**<0.001**
Cross-sectional	O3	Obesity	1.364625	**<0.001**	**<0.001**
Longitudinal	PM2.5	Incident CVD	1.091722	**0.005**	**0.009**
Longitudinal	PM10	Incident CVD	1.068815	**<0.001**	**<0.001**
Longitudinal	NO2	Incident CVD	1.115018	**0.047**	0.062
Longitudinal	O3	Incident CVD	1.084427	0.308	0.308

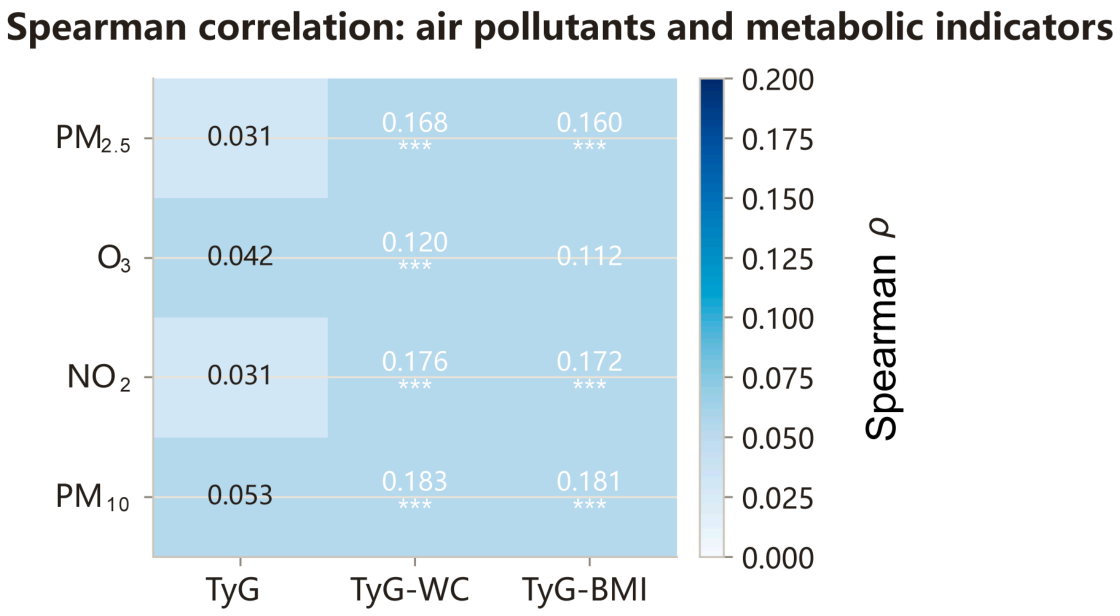

Figure S3. Spearman correlation analysis of air pollutant levels and key metabolic indicators. TyG, Triglyceride-Glucose Index; WC, waist circumference; BMI, body mass index.

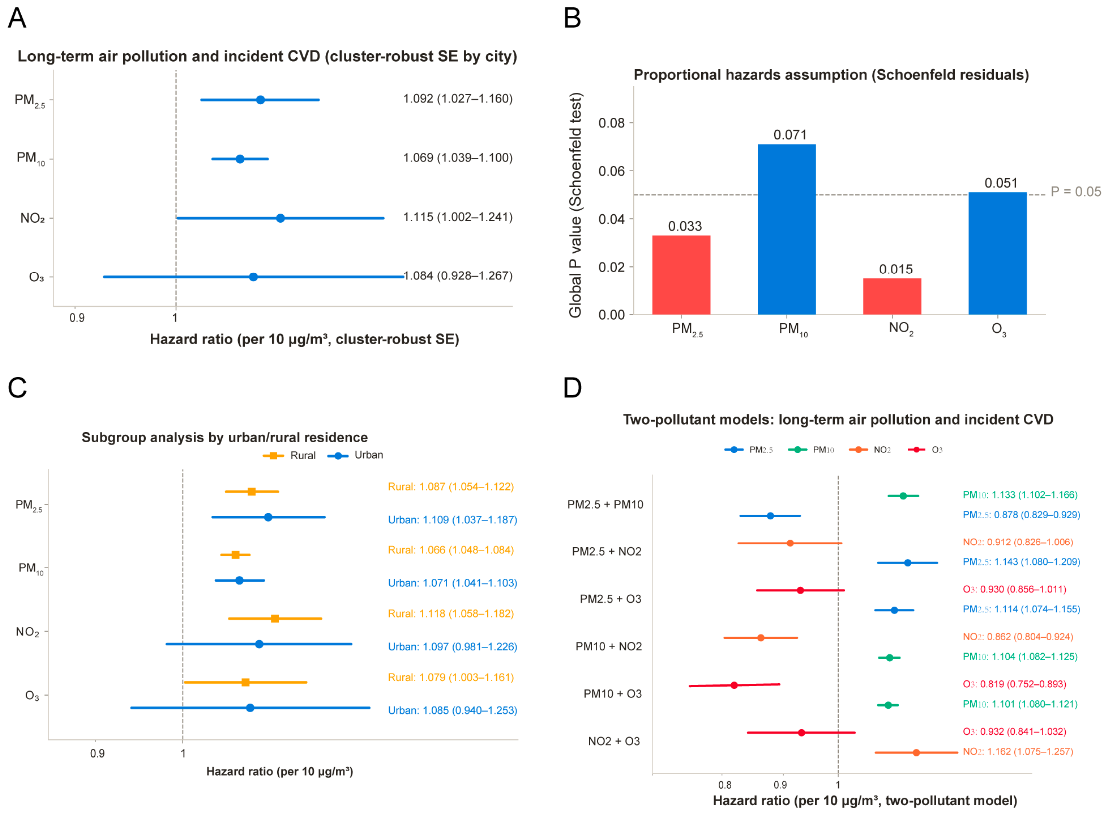

Figure S4. Association between long-term exposure to $PM_{2.5}$, PM_{10}, NO_2, and O_3 and incident CVD. (A) Hazard ratios per 10 μg/m³ increase from Cox proportional hazards models with cluster-robust standard errors (clustered by city). (B) Global P values from Schoenfeld residual tests for the proportional hazards assumption. (C) Subgroup analysis stratified by urban versus rural residence, with HRs and 95% CIs for each pollutant. (D) Two-pollutant

models with mutual adjustment for each pollutant pair.
